# Study on the Mechanism of *Dictyophora duplicata* Polysaccharide in Reducing Depression-like Behavior in Mice

**DOI:** 10.3390/nu16213785

**Published:** 2024-11-04

**Authors:** Chenxi Yang, Jiaqi Chen, Jie Tang, Lanzhou Li, Yongfeng Zhang, Yu Li, Changchun Ruan, Chunyue Wang

**Affiliations:** 1 Engineering Research Center of Chinese Ministry of Education for Edible and Medicinal Fungi, School of Plant Protection, Jilin Agricultural University, Changchun 130118, China; yangchenxi@mails.jlau.edu.cn (C.Y.); chenjiaqi@mails.jlau.edu.cn (J.C.); lilanzhou@jlau.edu.cn (L.L.); zhangyongfeng@jlau.edu.cn (Y.Z.); liyu@jlau.edu.cn (Y.L.); 2 Sichuan Institute of Edible Fungi, Chendu 610066, China; biotang@163.com; 3 Tianjin Institute of Industrial Biotechnology, Chinese Academy of Sciences, Tianjin 300308, China; 4 National Center of Technology Innovation for Synthetic Biology, Tianjin 300308, China; 5 Jilin Province Technology Research Center of Biological Control Engineering, Jilin Province International Cooperation Key Laboratory for Biological Control of Agricultural Pests, Changchun 130118, China

**Keywords:** *Dictyophora duplicata*, polysaccharide, depression, glutamate system, BDNF-TrkB-mTOR

## Abstract

Background/Objectives: Depression is a prevalent worldwide mental health disorder that inflicts significant harm to individuals and society. *Dictyophora duplicata* is an edible fungus that contains a variety of nutrients, including polysaccharides. This study aims to investigate the monosaccharide composition and molecular weight of the *Dictyophora duplicata* polysaccharide (DDP-B1), followed by an exploration of its antidepressant effects in chronic unpredictable mild stress (CUMS) mice. Methods: *Dictyophora duplicata* was purified using a DEAE-52 column and an S-400 column to obtain DDP-B1. The monosaccharide composition and molecular weight of DDP-B1 were investigated via high-performance gel permeation chromatograph. Six-week-old C57BL/6 male mice were utilized for the CUMS modeling to evaluate the antidepressant efficacy of DDP-B1. Fluoxetine served as the positive control group. The depressive-like behaviors and brain pathology of mice were evaluated. Immunofluorescence (IF) staining, metabolomics analysis, and western blot were employed to further investigate the underlying mechanisms. Results: DDP-B1 significantly alleviated the depression-like behavior of CUMS mice and increased the expression of SYN and PSD-95 in the mice’s brains, which was further validated by western blot. Metabolomics analysis indicated a reduction in serum glutamate in CUMS mice following DDP-B1 treatment. Moreover, DDP-B1 treatment led to an increase in levels of GABA_A_R, BDNF, p-TrkB and p-p70S6K. Conclusions: DDP-B1 regulated abnormalities in the glutamatergic system, subsequently activated the BDNF-TrkB-mTOR pathway and mitigated the pathological manifestations of CUMS mice. This study validated the potential of DDP-B1 as an antidepressant medication and established a theoretical foundation for the development of fungi with similar properties.

## 1. Introduction

Depression is a global psychiatric condition characterized by enduring feelings of sadness and anhedonia [1], leading to significant impairment in daily functioning and imposing a substantial burden on both society and the economy [2]. The pathogenesis of depression is intricate and multifaceted, with no single mechanism accounting for all aspects of the disorder. Common pathogenic mechanisms include the monoamine hypothesis [3], inflammation [4], the hypothalamic-pituitary-adrenal axis [5], a dysfunction of the glutamate system [6] and imbalances in the neurotransmitter and neurotrophic systems [7].

Most first-line antidepressant medications are primarily studied in relation to the monoamine neurotransmitter system. They achieve their antidepressant effects by blocking monoamine metabolism or reuptake [8]. However, there are still a considerable number of patients who fail to experience significant improvement within an appropriate timeframe [9]. Some patients even experience severe side effects such as nausea, diarrhea and insomnia [10]. Hence, it is of utmost importance to investigate safe and efficacious pharmacological treatments for depression and their underlying mechanisms.

Synaptic function is regarded as a potential crucial factor influencing mood in depression [11]. Synaptic damage serves as the primary pathological basis of this condition [12]. Glutamate is crucial for regulating synaptic plasticity and maintaining nervous system homeostasis and function [13]. Elevated levels of glutamate in the blood have been reported in depressed patients [14]. Gamma-aminobutyric acid (GABA) has been shown to play a regulatory role in glutamate levels. A reduction in GABA expression is associated with an increase in extracellular glutamate concentrations [15]. The GABAergic transmission mechanism synergistically interacts with the neurotrophic pathway of BDNF/TrkB. GABA_A_R-mediated depolarization in immature neurons enhances BDNF release, which subsequently stabilizes the endocytosis of GABAARs at the plasma membrane [16]. Patients with depression have been shown to have a decreased expression of tyrosine receptor kinase B (TrkB) and peripheral BDNF [17], which contributes to symptoms such as fatigue and cognitive impairment [18,19]. The release and binding of BDNF and its receptor TrkB activate the mammalian target of rapamycin (mTOR) pathway [20]. The activation of the mTOR signaling pathway promotes the synthesis of synaptic proteins, thereby multiplying the amount and functional performance of synapses [21].

*Dictyophora duplicata* is an edible fungus that contains a wide array of nutrients, including polysaccharides [22]. Polysaccharides are biomolecules known for their low toxicity and multiple biological functions [23]. *Dictyophora indusiata* polysaccharide has demonstrated an ability to promote neurite growth, modulate synaptic signaling and reduce synaptic dysfunction [24]. *Dictyophora echinovolvata* polysaccharide inhibits oxidative stress and improves neuronal apoptosis in the central nervous system [25]. *Dictyophora duplicata* is classified within the *Dictyophora* genus, and polysaccharides are one of its main components [26]. However, there have been no reports on the polysaccharides from the fruiting body of *Dictyophora duplicata* and their effect on depression.

In this study, *Dictyophora duplicata* polysaccharide (defined as DDP-B1) was extracted and purified from *Dictyophora duplicata*. The molecular weight (Mw) and monosaccharide composition were analyzed. The potential antidepressant effect of DDP-B1 was investigated in chronic unpredictable mild stress (CUMS) mice. To elucidate the mechanism of DDP-B1, non-targeted metabolomics, immunofluorescence (IF) staining and western blot were employed. The findings on DDP-B1 indicate that it may serve as a supplementary or alternative therapy for the treatment of depression.

## 2. Materials and Methods

### 2.1. Preparation of DDP-B1

**Figure 1:**
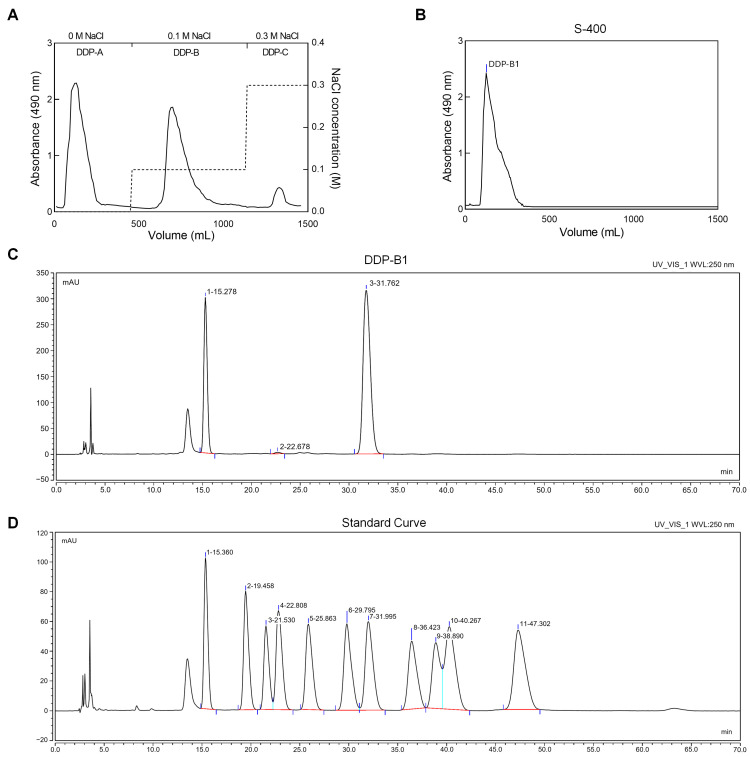
Purification of DDP-B1 and analysis of monosaccharide composition. (**A**) DDP was isolated and purified using a DEAE-52 column, with elution performed using 0, 0.1, and 0.3 M NaCl solutions. The fraction DDP-B was obtained by eluting with the 0.1 M NaCl solution. (**B**) DDP-B1 was purified utilizing an S-400 column. (**C**) Monosaccharide composition of DDP-B1. DDP-B1 was determined to consist of the monosaccharide 1 mannose (15.278 min), 2 glucuronic acid (22.678 min) and 3 glucose (31.762 min). (**D**) Monosaccharide standard curve. According to the retention time, they are 1 mannose (15.360 min), 2 glucosamine hydrochloride (19.458 min), 3 rhamnose (21.530 min), 4 glucuronic acid (22.808 min), 5 galacturonic acid (25.862 min), 6 D-galactosamine hydrochloride (29.795 min), 7 glucose (31.995), 8 galactose (36.423 min), 9 xylose (38.890 min), 10 L-arabinose (40.267 min) and 11 fucose (47.302 min). Black line: response value; red line: quantitative baseline; dark blue line: retention time; light blue line: division line.

The dried powder of *Dictyophora duplicata* was extracted with distilled water at 80 °C, following the procedure outlined in a previous study [27]. The solution was combined with ethanol to achieve an 80% concentration. The resulting mixture reacted at 4 °C for 24 h. Subsequently, centrifugation was performed at 6000 r/min for 10 min to obtain a precipitate, which was then dissolved and subjected to protein removal using the Sevage method. Small molecular impurities were removed through dialysis, after which the crude polysaccharide (DDP) was obtained via freeze-drying. DDP was then dissolved and subsequently purified with a Diethylaminoethyl Cellulose 52 (DEAE-52) column (C8930, Solarbio, Beijing, China) and eluted with a 0, 0.1 and 0.3 M NaCl solution. The polysaccharide components with uniform charge were separated (defined as DDP-A, DDP-B and DDP-C) (Figure 1A). HiPrep Sephacryl S-400 High resolution (S-400) columns (28935605, Cytiva, Washington, DC, USA) were employed for the further purification of DDP-B. Ultimately, DDP-B1 with uniform molecular weight was obtained for subsequent experiments (Figure 1B).

### 2.2. Mw Determination of DDP-B1

A high-performance gel permeation chromatograph (Waters 1515, Waters, Milford, MA, USA) was used to determine the Mw of DDP-B1. This analysis employed a Waters oscillometric detector (Waters 2410, Waters) in conjunction with a polymer matrix water-soluble size exclusion chromatography column (Shodex, Tokyo, Japan) measuring 8 × 300 mm. 1 mL of 0.05 M NaCl was added to DDP-B1 and subsequently eluted with 0.05 M NaCl at a flow rate of 0.65 mL/min, maintained at 40 °C. The molecular weight of DDP-B1 was determined by a dextran standard (analytically pure, Sigma-Aldrich, St. Louis, MO, USA).

### 2.3. Monosaccharide Composition Analysis

According to the prior study [27], 5 mg of DDP-B1 was subjected to hydrolysis with 2 M trifluoroacetic acid at 121 °C for 2 h. Methanol was utilized for washing, followed by blow-drying. The resulting product was then dissolved in sterile water, and the monosaccharide composition was analyzed using a Thermo U3000 liquid chromatography system (Thermo, Waltham, MA, USA). The chromatographic column used was the ZORBAX Eclipse XDB-C18 (Agilent, Santa Clara, CA, USA). The mobile phase was composed of equal volumes of acetonitrile and phosphate buffer, delivered at 0.8 mL/min, with detection conducted at 250 nm. The standard mixtures of monosaccharides utilized in this study included fucose, rhamnose, arabinose, galactose, glucose, xylose, mannose, glucosamine hydrochloride, galactosamine hydrochloride, galacturonic acid and glucuronic acid. All monosaccharide standards were obtained from Shanghai yuanye (analytically pure).

### 2.4. Animals and Treatment

Thirty-six male C57BL/6J mice (6 weeks old, 18–20 g, SPF) were obtained from the Experimental Animal Centre of Jilin Agricultural University. All animal experiments were approved by the Experimental Animal Welfare and Ethics Committee of Jilin Agricultural University (NO. 2023 08 24 003, approval date: 24 August 2023) and complied with the ARRIVE guidelines. The mice were given a week to acclimate to their new surroundings 1 week and maintained at a 12 h light/dark cycle at 22 ± 2 °C and 50 ± 10% humidity. The animals had unrestricted access to food and water prior to the establishment of the experimental model. 

According to the article in nature protocol [28], the mice were randomly assigned to four groups: the control group (Ctrl), the model group (CUMS), the fluoxetine (Flx)-treated group (CUMS + Flx) and the DDP-B1-treated group (CUMS + DDP-B1), with nine mice in each group. No more than five mice were housed per cage. Over a period of 12 weeks, the CUMS mice were subjected to one stressor per day. These stressors encompassed food deprivation (24 h), an inversion of the day/night cycle (24 h), restraint stress (2 h), tail suspension (10 min), hot stress (10 min), swimming in 4 °C cold water (5 min), forced swimming (5 min) and wet bedding (24 h). After 8 weeks of modeling, the CUMS mice were assessed using the sucrose preference test (SPT). At week 9, Ctrl and CUMS mice were given distilled water, CUMS + Flx mice were given 3 mg/kg Flx, and CUMS + DDP-B1 mice were given 100 mg/kg DDP-B1. After a 4-week treatment period, behavioral assessments were conducted to evaluate depression-like behaviors in mice. The data from behavioral tests were analyzed by an evaluator who was unaware of the treatment conditions.

The general health status of the mice was closely monitored throughout the entire experimental period. Following the completion of behavioral tests, all mice were euthanized using CO_2_ anesthesia. CO_2_ was displaced into the euthanasia vessel at a flow rate of 40% of the chamber’s volume per minute. Blood was collected from the tail vein and centrifuged at 3000 r/min for 10 min after standing, and the supernatant was centrifuged twice to collect serum. Collected organs including the brain, heart, liver, spleen and kidneys. The tissues were divided into two sections: one section was snap frozen in liquid nitrogen and then stored at −80 °C for future experiments, while the other section was fixed in 4% paraformaldehyde solution for histological analysis. The organ index was calculated using the following formula: organ index (%) = (weight of organ (g)/body weight (g)) × 100%.

### 2.5. Behavioral Tests

#### 2.5.1. SPT

Anhedonia is a feature of depression, usually assessed by the rate of sucrose consumption [29]. In accordance with the methodology of the previous study [30], prior to the commencement of formal testing, the mice were individually housed in cages and provided with adaptive training. This procedure involved providing two bottles of 1% sucrose solution for 24 h. Following this, one bottle was replaced with pure water for 24 h. The positions of the bottles were then exchanged every 12 h throughout the experiment. Prior to SPT, mice were fasted for 24 h following adaptation training. The sucrose preference rate in 24 h was calculated as follows: Sucrose preference rate (%) = (sucrose solution consumption/total liquid consumption) × 100%.

#### 2.5.2. Open Field Test (OFT)

Consistent with the prior research [31], mice were positioned at the periphery of an open field (50 cm × 50 cm × 40 cm) to allow for free exploration. The mice were acclimatized to the indoor environment before undergoing the OFT. The distances within the central area over 5 min were recorded and analyzed using the OFT device (OFT-100, Chengdu Techman, Chengdu, China).

#### 2.5.3. Tail Suspension Test (TST)

Similar to the previous study [32], mice were individually suspended from the tail with tape 2 cm from the tail tip for 6 min. A plastic tube was used to cover the tail to prevent mice climbing on it. The duration of immobility during the final 4 min of mice suspension was recorded utilizing the digbehv animal behavior analysis system (JLBehv-FSG-4, Shanghai Jiliang Shanghai, China).

#### 2.5.4. Forced Swimming Test (FST)

In accordance with the methodology of the previous study [32], mice were individually placed in a plexiglass cylinder (10 cm × 10 cm × 25 cm) filled with water. Each test session lasted 6 min, during which the time the mice remained motionless in the water for the last 4 min was recorded and analyzed using the digbehv animal behavior analysis system. Immobility is defined as the absence of any movement, except for that which is necessary to keep the head above water.

### 2.6. Hematoxylin and Eosin (H&E) Staining

Heart, liver, spleen, kidney and brain were preserved in 4% paraformaldehyde. The fixed tissues were subjected to dehydration, embedded in paraffin, processed through an ethanol gradient and subsequently sectioned at 4 μm slices. The staining was conducted according to this study [33].

### 2.7. IF Staining

As previously mentioned [33], the hippocampal sections were treated with 10% goat serum and incubated at 25 °C for 30 min. This was followed by incubation with primary and secondary antibodies, as detailed in Table S1. A panoramic scanning microscope (3DHISTECH CaseViewer, Budapest, Hungary) was employed for observation, while Image Pro Plus 6.0 was utilized for semi-quantitative analysis.

### 2.8. Non-Targeted Metabolomics Analysis

Serum samples were analyzed by non-targeted metabolomics based on ultra-high-performance liquid chromatography-tandem mass spectrometry, as previously described [34]. Venn analysis revealed the quantities of shared and distinct differential metabolites among the pairwise comparison groups. Heat map analysis was employed to depict the relative significance of metabolites as indicators of metabolic status. Kyoto Encyclopedia of Genes and Genomes (KEGG) was used to analyze the signal transduction pathways of the metabolites with significant differential expression in the brain.

### 2.9. Western Blot

Similar to previous studies [35], the proteins within the mice brain were extracted, and the concentrations of each group were homogenized. 40 μg of protein was separated using SDS-PAGE and subsequently transferred to a polyvinylidene fluoride membrane (0.45 μm) (10600023, Cytiva). After blocking with 5% bovine serum albumin for 6 h at 4 °C, the bands were incubated overnight with primary antibodies, followed by 4 h incubation with secondary antibodies (Table S1). Finally, protein bands were imaged using ultra-high sensitivity-enhanced chemiluminescence kits (GK10008; GlpBio, Montclair, CA, USA) via a chemiluminescence image analysis system (Monad, Wuhan, China). Quantitation was accomplished by employing ImageJ 6.0 software.

### 2.10. Statistical Analysis

Statistical analyses were performed using SPSS Statistics version 26 (IBMCorp., Armonk, NY, USA). GraphPad Prism 9.0 (GraphPad, Boston, MA, USA) was used to draw graphs. A one-way analysis of variance (ANOVA) followed by Tukey’s post hoc test was performed. Statistical significance was set at *p* < 0.05.

## 3. Results

### 3.1. Purification and Composition Analysis of DDP-B1

In this study, DDP-B1 is a polysaccharide obtained from *Dictyophora duplicata*. DDP-B1 was determined to consist of the monosaccharide glucose (66.56%), mannose (32.85%) and glucuronic acid (0.59%) (Figure 1C and Table S2), combined with the curve of the monosaccharide standard (Figure 1D). The peak molecular weight (Mp) of DDP-B1 was determined to be 6.63 × 10^6^ Da. The Mw of DDP-B1 measured at 1.08 × 10^7^ Da. The number-average molecular weight (Mn) of DDP-B1 was found to be 5.47 × 10^6^ Da (Table 1).

### 3.2. Effects of DDP-B1 Treatment on Depressive-Like Behavior in CUMS Mice

**Figure 2:**
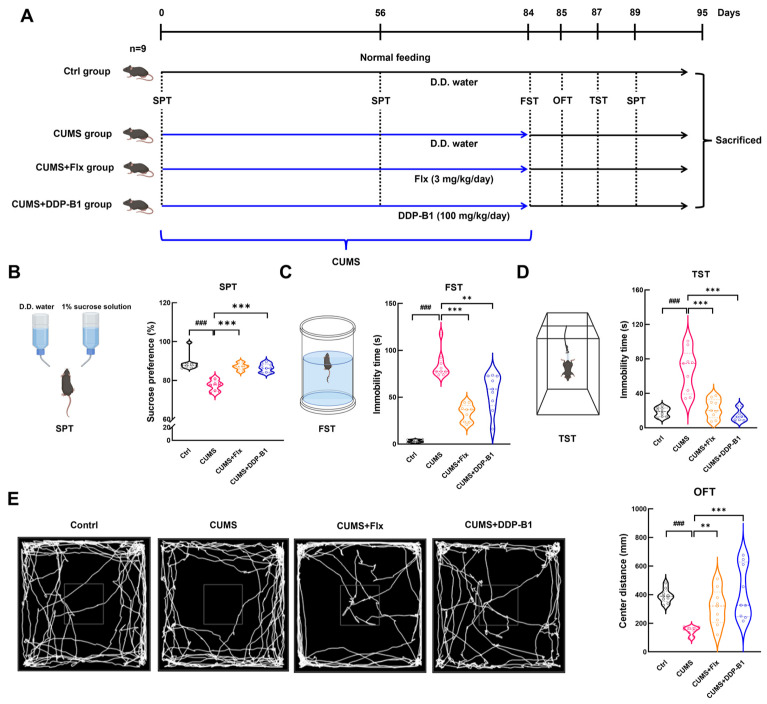
DDP-B1 alleviated CUMS-induced depression-like behaviors. (**A**) A schematic representation of the mice experimental procedure. After 8 weeks of modeling, mice were randomly divided into four groups as follows: Control, CUMS, CUMS + Flx, and CUMS + DDP-B1. (**B**) SPT. (**C**) FST. (**D**) TST. (**E**) OFT. Data were expressed as mean ± S.E.M. (*n* = 9). ^###^ *p* < 0.001 vs. Ctrl mice; ** *p* < 0.01, *** *p* < 0.001 vs. CUMS mice.

To investigate the mechanisms underlying the antidepressant effects of DDP-B1, mice were exposed to one stressor per day for 12 weeks, and treatment was given starting at week 9. To elucidate the antidepressant effects of DDP-B1, mice were administered DDP-B1 (100 mg/kg, gavage) and Flx (3 mg/kg, gavage) for 4 weeks (Figure 2A). The CUMS group demonstrated significant depression-like behaviors compared to the Ctrl group, as indicated by a marked decrease in sucrose preference during the SPT (*p* < 0.001) (Figure 2B). Additionally, this group exhibited significantly prolonged immobility times in both the FST (*p* < 0.001) (Figure 2C) and the TST (*p* < 0.001) (Figure 2D). The moving distance in the OFT’s center region was noticeably shorter (*p* < 0.001) (Figure 2E). These findings indicate that mice subjected to CUMS exhibit behaviors characteristic of depression. Compared to the CUMS group, mice treated with CUMS + DDP-B1 exhibited a significantly enhanced preference for sucrose (*p* < 0.001) (Figure 2B) and a reduction in immobility time during the FST (*p* = 0.003) (Figure 2C) and TST (*p* < 0.001) (Figure 2D). Additionally, there was a marked increase in the crossing distance within the central region of the OFT (*p* < 0.001) (Figure 2E). These findings indicate that DDP-B1 treatment effectively alleviated depression-like behaviors in mice. No statistically significant differences were detected in organ indices (heart, liver, spleen, kidney and brain) or histopathological analysis among the groups of mice (Figure S1). This indicates that DDP-B1 treatment does not exert a substantial burden on the organs of mice.

### 3.3. DDP-B1 Mitigates Pathological Conditions in the Brain of CUMS Mice

**Figure 3:**
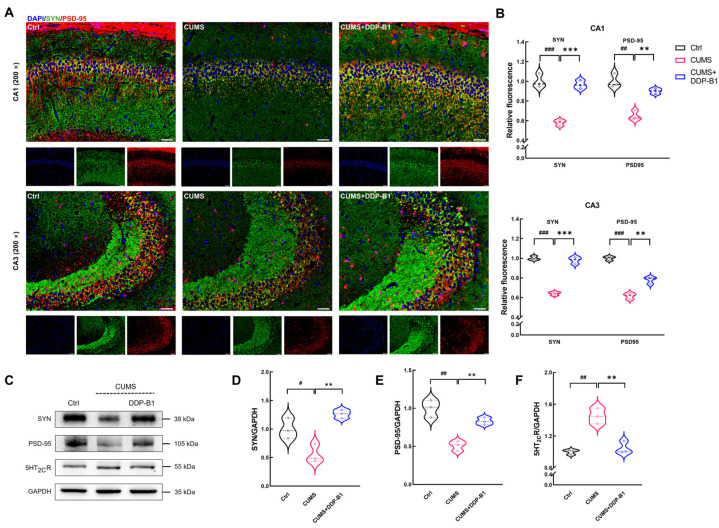
DDP-B1 mitigated synaptic damage in CUMS mice subjected to CUMS. (**A**) SYN and PSD-95 were analyzed using IF staining at a magnification of (200×; scale bar: 50 μm) in the CA1 and CA3 regions of the mice hippocampus. Blue: 4′,6-Diamidino-2′-phenylindole (DAPI); green: SYN; red: PSD-95. (**B**) Quantitative analysis for SYN and PSD-95 in IF staining. (**C**) Representative images of western blot for SYN, PSD-95 and 5HT_2C_R proteins in the mice. Results of quantitative analysis for the expression levels of (**D**) SYN, (**E**) PSD-95 and (**F**) 5HT_2C_R. Data were expressed as mean ± S.E.M. (*n* = 3). ^#^ *p* < 0.05, ^##^ *p* < 0.01, ^###^ *p* < 0.001 vs. Ctrl mice; ** *p* < 0.01, *** *p* < 0.001 vs. CUMS mice.

Hippocampal synaptic damage is considered to be the main pathological basis of depression [11]. The presynaptic membrane protein synaptophysin (SYN), postsynaptic density protein-95 (PSD-95) [36] and serotonin 2C receptors (5HT_2C_R) are closely related to changes in synaptic plasticity [37]. The impact of DDP-B1 on synaptic function and alterations in synaptic plasticity was confirmed through the implementation of IF staining. The findings indicated a decreased expression of SYN (*p* < 0.001) and PSD-95 (*p* = 0.002 in cornu ammonis (CA) 1, *p* < 0.001 in CA3) in the CA1 and CA3 subregions of the hippocampus in the CUMS group (Figure 3A,B). In contrast, the expression levels of both proteins (*p* < 0.001 of SYN, *p* = 0.001 of PSD95 in CA1, *p* = 0.004 of PSD95 in CA3) were significantly upregulated in the CUMS + DDP-B1 mice (Figure 3A,B). To further substantiate the findings, western blot was employed to confirm that DDP-B1 enhanced the expression levels of SYN (*p* = 0.002), PSD-95 (*p* = 0.002) and 5HT_2C_R (*p* = 0.005) in mice brains (Figure 3C–F). These findings indicated that DDP-B1 effectively mitigated pathology associated with depression.

### 3.4. DDP-B1 Regulated Serum Metabolites in CUMS Mice

**Figure 4:**
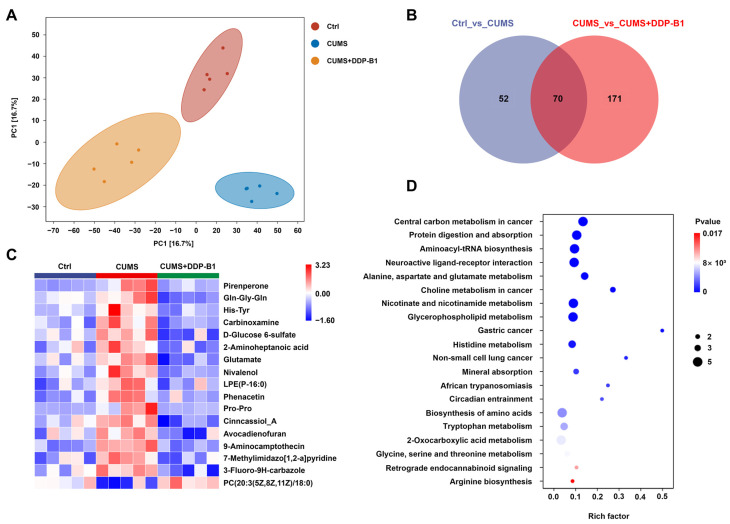
Metabolomics analysis of mouse serum. (**A**) OPLS-DA score analysis. (**B**) Venn analysis. (**C**) Heatmap of significantly altered metabolites among Ctrl, CUMS and CUMS + DDP-B1 groups. (**D**) KEGG enrichment pathway diagram (*n* = 5).

To further explore the mechanisms by which DDP-B1 alleviates depression, this study conducted an analysis of metabolite levels in mouse serum utilizing untargeted metabolomics. Orthogonal partial least-squares discriminant analysis (OPLS-DA) revealed that the effect of DDP-B1 on metabolites significantly differs from that observed in the CUMS group, aligning more closely with the Ctrl group (Figure 4A). The Venn diagram indicated that, in comparison to Ctrl mice, 122 metabolites exhibited significant expression differences in the CUMS mice. When compared to the CUMS + DDP-B1 mice, 241 metabolites showed significant expression differences. Among them, 70 differences were identified in all mice (Figure 4B). Compared to the Ctrl group, heatmap analysis indicated that 16 metabolites, including glutamate, were upregulated and one metabolite downregulated in the CUMS group, while the levels of the above metabolites were reversed after DDP-B1 treatment (Figure 4C). In established studies, it has been observed that serum glutamate levels are elevated in patients with depression and tend to decrease following treatment [14]. The KEGG pathway enrichment analysis revealed significant enrichment in the alanine, aspartate and glutamate metabolism pathways (Figure 4D). The integrated metabonomic data indicated that the glutamatergic system may serve as a mechanism through which DDP-B1 exerts its antidepressant effects in CUMS mice.

### 3.5. DDP-B1 Improved BDNF-TrkB-mTOR Signaling Pathway in the Brain

**Figure 5:**
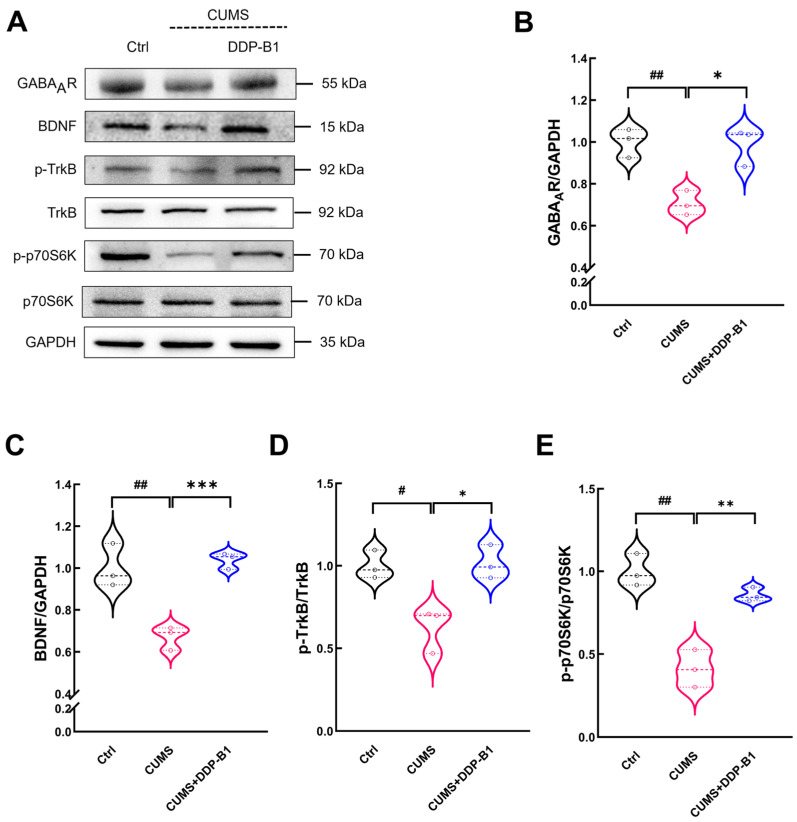
DDP-B1 modulates the BDNF-TrkB-mTOR signaling pathway. (**A**) Representative images of western blot. The administration of DDP-B1 significantly enhanced the expression levels of (**B**) GABA_A_R, (**C**) BDNF, (**D**) p-TrkB and (**E**) p-p70S6K in the brains of CUMS mice. Data were expressed as mean ± S.E.M. (*n* = 3). ^#^ *p* < 0.05, ^##^ *p* < 0.01 vs. Ctrl mice; * *p* < 0.05, ** *p* < 0.01, *** *p* < 0.001 vs. CUMS mice.

The results indicated that CUMS significantly impacted the glutamatergic system, leading to a marked reduction in the protein levels of GABA_A_R (*p* = 0.005) in the brains of mice. Furthermore, CUMS significantly decreased the expression levels of BDNF (*p* = 0.009), phosphorylated TrkB (p-TrkB) (*p* = 0.015) and phosphorylated p70 ribosomal S6 kinase (p-p70S6K) (*p* = 0.002) in comparison to the Ctrl mice. Compared to the CUMS group, DDP-B1 treatment significantly enhanced the expression levels of GABA_A_R (*p* = 0.011), BDNF (*p* < 0.001), p-TrkB (*p* = 0.016) and p-p70S6K (*p* = 0.003) (Figure 5A–E. This finding provided further evidence that DDP-B1 may regulate the BDNF-TrkB-mTOR signaling pathway by regulating the glutamate system, ultimately affecting synaptic regulation and alleviating depression.

## 4. Discussion

More and more research has established a robust correlation between synaptic function and both mood and cognitive processes in individuals diagnosed with depression [38]. Synaptic damage is the primary pathological basis of depression [13]. The maintenance of synaptic plasticity homeostasis represents a crucial regulatory factor in the treatment of depression [11], as it is associated with the capacity to perceive, evaluate and store complex information, as well as to respond adaptively to external stimuli [39,40]. It can be reasonably inferred that the regulation of synaptic plasticity holds significant potential for alleviating depression. DDP-B1 is a natural extract from *Dictyophora duplicata* that offers the advantage of extremely low toxic side effects [41]. Its Mw was found to be 1.08 × 10^7^ Da, and its composition was determined to be 66.56% glucose, 32.85% mannose and 0.59% glucuronic acid. In studies of *Dictyophora* polysaccharides, it was shown to promote neurite growth and reduce synaptic dysfunction by regulating the fatty acid catabolism, amino acid biosynthesis and phenylalanine metabolism pathways, thereby reducing nerve damage [24]. In addition, *Dictyophora echinovolvata* polysaccharide plays a role in preventing neuronal apoptosis by improving oxidative stress [25]. In this study, we showed that DDP-B1 treatment attenuated depressive-like behavior in mice. Depression is frequently associated with synaptic plasticity defects and neurogenic damage [42], which are associated with emotional disorders and spatial learning and memory disorders [43]. SYN is the marker of synaptic development and activity. A reduced expression of SYN results in a decrease in synaptic vesicles, an excessive pruning of synapses and impaired synaptic plasticity [44]. PSD-95 is an important factor in synaptic plasticity and postsynaptic membrane stabilization [45]. It ensures correct synaptic size, shape and function by rearranging the mass and quantity of synaptic spines [46]. CUMS was shown to decrease both the quantity and the functionality of spinous synapses in pyramidal cells, as well as to reduce the expression levels of PSD-95 and SYN [15]. 5HT_2C_R are extensively distributed across cortical limbic structures [47]. The levels of 5-HT_2C_R in the ventral hippocampus and frontal cortex of rats were observed to increase under depressive conditions [48]. The decreased expression of 5HT_2C_R increases the volume and number of pyramidal neurons and improves the adaptive ability of the hippocampus [49]. In this study, DDP-B1 treatment increased levels of SYN and PSD-95 and decreased the level of 5HT_2C_R, which suggests that DDP-B1 exerts a beneficial effect on depressive symptoms by regulating synaptic plasticity.

Studies have shown that plasma glutamate level was significantly increased in patients with depression and decreased after treatment [14]. This is consistent with our results. Glutamate serves as a metabolic precursor of GABA [50]. GABAergic neurons are equipped with glutamate decarboxylase, an enzyme that facilitates the conversion of glutamate into GABA [15]. Chronic stress results in a reduction in GABA expression [51], which ultimately contributes to elevated levels of extracellular glutamate [50]. GABA increases the phosphorylation of Ser133 on CREB [52], further promoting the expression of BDNF [53,54]. BDNF has the ability, by binding to its receptor TrkB, to activate a number of downstream signaling pathways involved in the regulation of synaptic plasticity, including mTORC1 [55,56]. The mTOR synapses are able to modulate autophagy and protein translation in the hippocampus by regulating the downstream protein p70S6K, which in turn increases the levels of PSD-95 and SYN in CUMS, thereby attenuating CUMS-induced synapse loss [21]. In this study, DDP-B1 administration not only increased the protein expression levels of GABA_A_R and BDNF in CUMS mice, but also increased the protein expression levels of p-TrkB and p-p70S6K. These results indicate that DDP-B1 improves the abnormal glutamatergic cycle and BDNF-TrkB-mTOR signaling pathway in CUMS mice.

## 5. Conclusions

In conclusion, it was established through this study that the Mw of DDP-B1 is 1.08 × 10^7^ Da. Furthermore, its composition was determined to consist of 66.56% glucose, 32.85% mannose and 0.59% glucuronic acid. Meanwhile, the current study presents evidence that DDP-B1 exerted antidepressant effects by activating the BDNF-TrkB-mTOR pathway. These findings suggest that DDP-B1 may be a promising target for depression treatment. There are still some limitations to this study. Although the monosaccharide composition of DDP-B1 has been detected, the structural components with antidepressant effects need further research.

## Figures and Tables

**Table 1:** Analysis of molar mass moments of DDP-B1.

Mp (Da)	Mw (Da)	Mn (Da)
6,630,560	10,814,711	5,465,458

Mp: the peak molecular weight. Mw: molecular weight. Mn: the number-average molecular weight.

## Data Availability

The original contributions presented in the study are included in the article, further inquiries can be directed to the corresponding author.

## Supplementary Materials

The following supporting information can be downloaded at: https://www.mdpi.com/article/10.3390/nu16213785/s1, Table S1: Details of antibodies used in IF staining and western blot; Table S2: The monosaccharide composition of DDP-B1; Figure S1: Safety evaluation of CUMS mice treated with DDP-B1.

## Abbreviations

5HT_2C_R: serotonin 2C receptors; ANOVA: analysis of variance; BDNF: brain-derived neurotrophic factor; CA: cornu ammonis; CREB: cyclic adenosine monophosphate response element binding protein; CUMS: chronic unpredictable mild stress; DAPI: 4′,6-Diamidino-2′-phenylindole; DDP: *Dictyophora duplicata* polysaccharide; Flx: fluoxetine; FST: forced swimming test; GABA: gamma-aminobutyric acid; GABAR: GABA receptor; GABA_A_R: gamma-aminobutyric acid type A receptor; H&E: hematoxylin and eosin; IF: immunofluorescence; KEGG: Kyoto Encyclopedia of Genes and Genomes; Mn: number-average molecular weight; Mp: peak molecular weight; mTOR: mammalian target of rapamycin; Mw: molecular weight; OFT: open field test; OPLS-DA: orthogonal partial least-squares discriminant analysis; p70S6K: p70 ribosomal S6 kinase; PSD-95: postsynaptic density protein-95; p-TrkB: phosphorylated TrkB; p-p70S6K: phosphorylated p70 ribosomal S6 kinase; SDS-PAGE: sodium dodecyl sulfate-polyacrylamide gel electrophoresis; SPF: specific pathogen free; SPT: sucrose preference test; SYN: synaptophysin; TrkB: tyrosine receptor kinase B; TST: tail suspension test.
