# Supplementary material for: Study on the Mechanism of Dictyophora duplicata Polysaccharide in Reducing Depression-like Behavior in Mice
Source: Nutrients. 2024 Nov 4;16(21):3785. doi: 10.3390/nu16213785 (PMC11547661; doi:10.3390/nu16213785)
Supplement: Supplementary file 1 [file nutrients-16-03785-s001.zip › nutrients-3257981-supplementary.pdf]

Supplementary Data

Table S1. Details of antibodies used in IF staining and western blot.

**Antibody**	**Molecular weight**	**Catalog number**	**Dilution**	**Company**	**Area**	**adhibition**
SYN	38 kDa	AF0257	IF: 1:200<br>WB: 1:1000	Affinity	Cincinnati, OH, USA	IF, WB
PSD95	105 kDa	AF5283	IF: 1:200<br>WB: 1:1000	Affinity	Cincinnati, OH, USA	IF, WB
KPL conjugates anti-rabbit lgG (H+L)		5220-0336	1:400	SeraCare	Gaithersburg, MD, USA	IF
$5HT_{2C}R$	55 kDa	#DF3501	1:1000	Affinity	Cincinnati, OH, USA	WB
$GABA_AR$	55 kDa	DF6583	1:1000	Affinity	Cincinnati, OH, USA	WB
BDNF	15 kDa	#DF6387	1:1000	Affinity	Cincinnati, OH, USA	WB
TrkB	92 kDa	A12325	1:1000	ABclonal	Boston, MA, USA	WB
p-TrkB (Y705)	92 kDa	ab229908	1:1000	abcam	Cambridge, UK	WB
p70S6K	70 kDa	#AF6226	1:1000	Affinity	Cincinnati, OH, USA	WB
p-p70S6K	70 kDa	#AF3228	1:1000	Affinity	Cincinnati, OH, USA	WB
GAPDH	35 kDa	E-AB-40337	1:2000	Elabscience	Wuhan, China	WB
goat anti-rabbit		E-AB-1003	1:4000	Elabscience	Wuhan, China	WB

Table S2. The monosaccharide composition of DDP-B1.

No.	Monosaccharide	Content (%)
1	Glucose	66.56
2	Mannose	32.85
3	Glucuronic acid	0.59

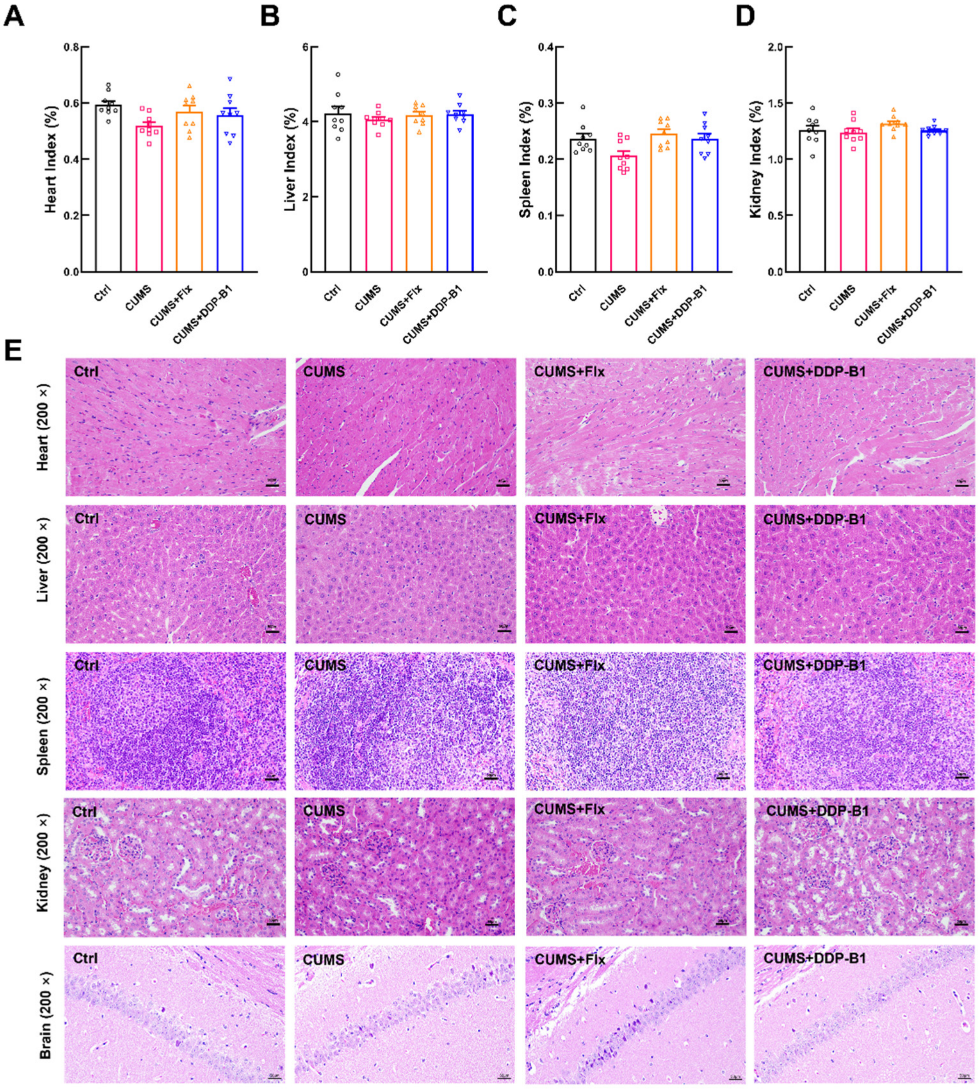

Figure S1. Safety evaluation of CUMS mice. (**A**) Heart index, (**B**) liver index, (**C**) spleen index and (**D**) kidney index (n = 9); (**E**) Representative images of H&E staining of the heart, liver, spleen, kidney and brain (200×; scale bar: 50 μm). Data were expressed as mean ± S.E.M.
